# Amputation and Dressings

**Published:** 1894-07

**Authors:** Howard J. Williams

**Affiliations:** Macon, Ga.


### AMPUTATION AND DRESSINGS.*

BY HOWARD J. WILLIAMS, A.M., M.D.,
MACON, GA.

Throughout the history of amputations, from the time when Hippocrates taught that amputation should ordinarily be limited to the removal of gangrenous portions of limb by cutting through dead tissues alone, and in compound fractures allow the dead portion to fall off of itself, down to the present day, when we anticipate the line of demarcation by early amputation high above the seat of gangrene, we trace many of the most important steps of surgery. From natural amputation by gangrene, the first step was to incisions through healthy tissues straight through the skin, muscles and bone alike with clippers and chisels, then the introduction of flaps to cover the bone. Ligation of arteries followed by the searing of blood-vessels with hot irons. The bloody amputation was done away with by the tourniquet, and now Esmarch's bandage gives us the bloodless operation. Anesthesia removes shock and agony; while pus, germ infection and tardy healing by granulation have yielded to primary union under aseptic methods. With improvement of methods came lessened mortality, until now the careful operator can count on an almost perfect record. The next advance will be, under antiseptics and antisepticism, a demand for fewer amputations and a larger saving of useful members. Traumatism, always the most fruitful feeder of the amputating table, is now being brought under control, and the indications for operation that

*Read before the Macon Medical Society. See page 296.

were formerly imperative are gradually being reduced in number. Asepsis has extended its influence not only to traumatic lesions, but has made it possible to remove diseased tissues that formerly led to amputation. Early wide excision of neoplasms, early resection of diseased bone, excision of necrotic tissues and washing out inflamed joints under aseptic methods tend to lower the demand for amputation. The successful surgeon no longer looks with pride upon the number of lives saved by amputation, but congratulates himself that he saves useful limbs.

The indications for amputation are various. Almost any injury or variety of disease affecting a limb may under certain circumstances call for the operation. What are some of the indications?

Deformities, either congenital or acquired, may, for the health or convenience of the individual, require amputation. In neglected cases of congenital club-foot, the substitution of an artificial for the deformed member may at times become necessary for the convenience and usefulness of the patient.

Three years ago Dr. McHatton and I advised amputation at the middle of the thigh of a young lad with a badly anchylosed knee-joint in preference to exsection of joint. The destruction of the bones and joint was so geat that the latter operation would have left an exceedingly short and useless limb. Deformities the result of cicatricial contractions may for convenience demand amputation. Some time ago I removed a contracted third finger of the right hand of a seamstress, the finger being constantly in the way of her pursuing her avocation. The contraction was the result of a badly treated palmar abscess. Painful cicatricial union of mangled extremities, if nerve stretching, nerve section, etc., gives no relief, will often require the operation for the health and usefulness of the individual. Some years ago I advised the removal of a hand of a man who had been badly mangled in machinery. Nerve stretching and section had been tried without relieving the pain before I saw him. He at the time declined the operation, but afterwards, in another city, submitted to it. In this class of cases the operation is one of expediency and should only be performed after it has been determined that the higher surgical procedures, such as exsection and resection, are impracticable, and only under circumstances as regard the age and condition of the patient which would promise a favorable termination of the case.

In malignant and morbid growths of the joints and extremities the leaning of surgical opinion is still to amputation, though early wide removal of morbid growths, exsection and resection is finding favor. Life in these cases is prolonged by amputation with the hope of non-recurrence; should recurrence, however, take place, relief from pain, ease of mind and a fair degree of health is obtained, while the fatal termination is delayed perhaps one, two, three or more years. In two cases of sarcoma that have come under my personal observation, one year in one and five years in another, was added to the life of each individual. In May, 1892, I amputated, in a case of sarcoma of the foot, just below the knee; in May of this year the patient died with recurrence in the chest-wall. Had not the operation been performed, he would have died within sixty days; and had he submitted to the operation six months earlier, when I first urged him to consent, I believe recurrence would have been delayed to a later period. In the other case, three amputations were performed, first of the foot with freedom from recurrence for two years, the second amputation below the knee with two years' immunity; the last operation at the upper third of the thigh, performed by Dr. McHatton, was followed by recurrence in the pleural cavity in one year, the patient dying one year and six months after the last amputation.

In various diseases not malignant of the bones and joints necrosis, caries, destructive arthritis of syphilitic or tubercular origin, amputation is necessary, provided better results cannot be obtained by more conservative methods. Here I would particularly urge the careful consideration of resection and exsection, before subjecting a patient to the complete loss of a limb. In two amputations for necrosis in children of seven and eight years, one of the foot beginning in the astragalus following a wound from a carpet tack, the other of the arm beginning in a neglected fracture of the humerus at the elbow, I believe I could have avoided amputation had I been allowed to perform conservative operations when I first saw the cases.

Amputation is demanded in gangrene or mortification from whatever cause, except dry gangrene of old persons having cardiac or other central lesions. Though the operation has been recommended by some bold operators, in these cases it is extremely hazardous; the condition of these old people is already so low that amputation promises nothing. Embolic gangrene of young people on the other hand is amenable to high amputation. In gangrene following intense inflammatory action, burns and scalds, frost-bite, and in mortification following low states of the system, such as typhoid fever, the rule is to wait for the formation of a line of demarcation between the living and the dead tissues. This rule should always be followed; if the surgeon cuts through tissues, the vitality of which is in doubt, there will not only be renewal of mortification in the flaps, but the patient is subjected to the shock of operation with the prospect of a secondary amputation if not a fatal termination. In gangrene of traumatic origin, on the other hand, such as gunshot wounds, injury to blood-vessels, compound fractures, severe mashed wounds of the soft parts and hospital gangrene, amputation should be performed early before the formation of a line of demarcation and well above the seat of the disease. Delay is fatal, there is great loss of strength and rapid exhaustion of the vital forces which can only be relieved by prompt action. I have amputated five times for traumatic gangrene with two fatal results. Both cases I believe due to the operation being too long delayed by the surgeon in attendance. In the other three cases the operations were performed early and successfully. There is no conservative procedure to take the place of amputation in gangrene.

The destructive effects of intense heat and cold, bites of animals and local destructive inflammation of chemicals, such as extensive sloughing destroying blood-vessels and secondary hemorrhage, and also for the later effects, as cicatricial contraction and malignant diseases of cicatrices, amputation is often required.

Various lesions of the blood-vessels require amputation, provided other conservative procedures cannot save life or limb. Laceration of the larger arteries, as the popliteal, if failure or impossibility to ligate the ends of the artery occur, demands the operation. In diffuse traumatic aneurism, either following a traumatism or due to rupture of a spontaneous aneurism, amputation may be necessary. Gangrene of a limb following deligation of an artery of course requires the operation. Two years ago I ligated the external iliac artery for circumscribed traumatic aneurism of the upper third of the femoral; gangrene of the limb followed, and had the patient's condition been favorable a secondary amputation, either high up in the thigh or at the hip-joint, would have been performed. Perhaps in this case primary amputation would have been better.

Among traumatic lesions there is only one class of cases in which conservative operations admit of no discussion—that is, avulsion or complete crushing or tearing off of a part of a limb; here only amputation can be performed; other lesions of this class do admit of some consideration with regard to conservative surgery. In extensive laceration and contusion of the soft tissues amputation is often required, but no doubt conservative antiseptic surgery may frequently save these limbs. Let the surgeon in these cases be very careful how he decides to operate. The most careful examination of the lacerated tissues is needed, the most minute weighing of the probable influence of the hygienic surroundings and the general condition of the patient, and the conservative possibilities of antiseptic methods should be considered before deciding to condemn a patient to the total loss of a limb. If the main blood supply is good, and there is left a good venous return circulation, if the hygienic surroundings are favorable, a good general condition of the patient present, the wound is cleaned and the patient is seen early, do not be in too great a haste to operate,—wait. Perhaps if the tissues can be replaced and sutured in their natural positions, good drainage set up and thorough antiseptic methods instituted with careful and minute attention to every detail in after treatment, good results may follow in the most unpromising case. I have seen such cases and recall with pleasure hands and feet saved by such careful waiting. On one occasion, however, I was forced to amputate in a case of this character by the surroundings of the patient and the want of skilled attention, but I felt that under other circumstances the limb could have been saved. I was called seventy-five miles away to operate on a severe anal fistula. While at the station, I was asked to see a man whose leg had been badly lacerated from the knee to the toes in an accident on a saw-mill railroad three hours before I arrived. The bones, joints, larger blood-vessels and some of the muscles were uninjured, but the skin and most of the muscles were badly torn. No dressing had been applied. The weather was hot, the shanty in which the patient lived was very septic and the doctor in attendance knew nothing of modern antiseptic methods. Only one thing could be done, amputate above the knee and instruct the doctor in the use of some simple antiseptics which I left with him.

In compound fractures and dislocations amputation is generally demanded, but under thorough antisepticism many cases may be saved. I look back with pleasure to a compound comminuted fracture of the femur just above the knee-joint, saved even though there remained a partial anchylosis of the joint. In this case, owing to the age of the patient, a man of sixty years, I was urged by an older practitioner to amputate, but after removing several small pieces of bone, I succeeded in saving the limb and life of the patient.

I recall what to me was a remarkable case of conservative surgery in the hands of Dr. Ferguson—a compound fracture of the humerus, which came into his hands ten days after injury, the bones exposed and the wound suppurating profusely; yet he resected the ends of the bone successfully, and the patient has now a useful arm. I have records of many injuries to the hand involving the bones and joints that have been saved by strict antisepticism and careful nursing, and recall three patients with good feet that have had severe compound comminuted fractures involving the ankle, one having compound fracture of both feet. Dr. McHatton has records of four compound fractures at the ankle saved from amputation by the same careful treatment. It is the rule in compound fractures involving the larger joints to amputate, yet many of these limbs may be saved by antiseptics and exsection ; if, however, the main artery is destroyed or badly injured it is impossible to save the limb, and amputation is demanded.

In gunshot wounds amputation is often indicated; bones shattered by conical balls, and soft tissues lacerated by small shot cutting off the blood supply are often so severe that other methods are useless.

If, however, the main arteries are not involved resection and exsection may often be successfully employed. Gunshot wounds of the joints almost always require amputation.

I have devoted a great deal of this paper to the indications for amputation, for the specific purpose of enforcing the fact that the higher conservative operations and strict antiseptic treatment may frequently be employed in place of amputation. It is the duty of every surgeon to be conservative, and as far as it is consistent with the saving of life, to do the utmost to save the limb of the patient.

Let us now consider the time when an amputation should be performed. This is of particular interest in operations for traumatic lesions; in chronic diseases, necrosis, caries, etc., and in morbid growths and deformities, only questions of the general condition of the patient and hygienic surroundings are involved. The operation is better borne in these diseases than in traumatic lesions, owing to the fact that the system is in better condition, provided general sepsis and exhaustion from disease have not taken place before the operation is undertaken, and there is no element of shock to contend with from previous injury. In traumatism, on the other hand, the presence of the shock throws the entire system into a disturbed condition and frequently the patient is exhausted by severe hemorrhage, these giving a high degree of danger not present in non-traumatic lesions.

For convenience of description, the time for operating in traumatism has been divided into three periods; first, *immediate*, that is just after an injury, or within the first two or three hours, while the patient is in the state of shock and exhausted by hemorrhage; second, *primary*, or after shock has passed off and reaction taken place and before inflammatory fever sets in, that is within the first twenty-four hours; and third, *secondary*, or in the period when inflammation usually sets in, any time after the first twenty-four hours.

The first period, or immediate, is by all means the most dangerous, and no amputation of any magnitude should ever be undertaken during this stage. To a patient in this period the surgeon who operates adds the additional dangers of anesthesia and shock of amputation to the nervous and mental shock from the sudden violent traumatism that crushes a limb, and to the exhaustion of hemorrhage. In these days of antiseptic detail in primary dressings delay is safe, and it is rank folly to rush a patient from one danger into another. In railroad surgery this is particularly true, for in this class of cases are to be found the most violent forms of shock. I have declined to operate in this period and will continue to do so. I have performed my share of operations for traumatisms, and have yet to record "a successful amputation but the patient died." The only deaths after amputation I have had were two, and they were for traumatic gangrene in consultation practice. It is my rule to wait for reaction, before performing any operation; and this waiting is not inaction but is work, controlling hemorrhage, assisting reaction by anodynes and stimulants, and rendering the wound and parts thoroughly antiseptic. After reaction has set in and the patient has fully entered the primary period then is the greater safety; and I believe it is the teaching and practice of the best surgeons to wait for it before operating. I believe that the time for amputating will yet be delayed to the secondary or imflammatory period, particularly for compound and comminuted fractures. Under antiseptic methods inflammatory action can be controlled, and the surgeon of the future will hold over his amputations with the hope that the part or limb may be saved. In several instances such has been my experience; starting out in a case believing that amputation would be necessary, I have found I could save the limb. That delay in amputations will be the rule in the future I believe will be particularly true in railway injuries. For in these injuries when the shock is so great and other complications are present, no harm can come from delay, but perhaps material aid can be found in it. It is the rule in railway amputations, for reasons to be presently given, to go high above the seat of injury, higher than in ordinary traumatisms, and no doubt the occurrence of a certain degree of traumatic inflammation following delay may help to locate the best point for operation.

The danger of amputation depends upon the point where an operation is performed; it increases as we approach the body and is greater in the lower than in the upper extremity. The danger also depends upon the character of the lesions; least dangerous, other things being equal, are amputations for deformities; then malignant and morbid growths; next diseases of bone and joints, if uncomplicated by sepsis and constitutional dyscrasia; while most dangerous of all are amputations for traumatisms. Children will bear amputation well, old persons poorly; sex has little influence; while hygienic surroundings and constitutional conditions exert an influence on the mortality.

The point at which an amputation should be performed is of great interest, and the cardinal rule is to save as much of the limb as possible consistent with the safety of the patient and securing a good, healthy stump. In amputations about the hand this is essentially important. It is surprising to see the amount of use that can be obtained even from the stumps of fingers. I have in my records the case of a carpenter who has only the proximal phalanges of the first and second fingers of his hand left, the thumb and other fingers entirely gone. He is an expert in picking up nails with what he calls his "nubbins" and handles his tools well. Another mechanic lost all of the hand except the little finger, while several patients have only a thumb, or a thumb and one finger. These persons become exceedingly expert in using their deformed hands and fingers, and are extremely grateful for conservative work in this line. It is astonishing, on the other hand, how awkward a person becomes in walking after losing the toes and portions of the foot. This is so great that I am of the opinion that Chopart's and Hays's amputations, or through the medio-tarsal articulation, should never be performed. In these operations the tendons that counteract the tendo-achillis are cut off, the foot is drawn downward, the individual walks clumsily on the end of the stump, and the attempt to adjust an artificial foot is an awkward failure. Symes' and Pirogoff's operations through the tibia-tarsal articulation are a little better, but the objection to these is the back part of the heel is turned downward and forward or under the end of the bone in forming the flaps, and the patient walks on tissues that were never intended to take the place of the tough fascia of the heel and foot. An artificial foot can be more easily adjusted, yet I doubt that any material advantage is gained. In injuries necessitating these operations I would advise amputation above the ankle at the junction of the middle and lower thirds of the leg, since all artificial supports after these operations is maintained on the side and head of the tibia and fibula, and on the thigh above, and good natural movement is obtained. Amputations in the joints are not so good as through the continuity of the bones, and the adjusting of a well fitting, graceful artificial limb is more easily accomplished in the latter than in the former operations. This question of adjusting artificial support after amputations in these days is one of importance, and we should lend every aid to the manufacture of artificial limbs, by careful study of prosthesis. We should acquire some knowledge of artificial limbs, learn the points of bearing that are taken in all kinds of amputations, and study the changes that take place in a stump after using an artificial, so that we can shape the character of our amputations to give the best results to the manufacturer's art.

There is one question in discussing the point of an amputation to which I wish to draw your attention. In the ordinary traumatisms, those resulting from falls, from bodies falling upon or being projected against a limb, or when a vehicle on the streets runs over a limb, etc., causing compound and comminuted fractures and amputation is indicated, the usual rule can be followed and the operation performed near the seat of injury, the vitality of the tissues being good, without the fear of subsequent sloughing or gangrene. In railroad injuries, on the other hand, such rule will not hold good. In these lesions the magnitude of the force and momentum is so great that the tissues are devitalized in both directions far beyond the point of contact. The extent of this destruction varies with the amount of force and degree of momentum. The muscles and blood-vessels bear the damage best, while the skin, subcutaneous tissues and bone suffer most. Soon after the injury the skin sloughs away and the bone becomes necrotic. If you amputate in this area you are bound to have sloughing of the flaps and a long siege of necrosis and then a secondary amputation. Surgical science has not yet discovered a means of knowing immediately after the accident to what extent this destruction will take place, but we do know it will occur. Hence it is now the cardinal rule in railway injuries due to crushing by moving trains to amputate high above the seat of injury. And for this reason I would urge postponing amputation until the secondary period in these lesions, waiting for inflammatory action, controlled by antisepticism, to aid in determining point of operation.

The selection of the method of amputation or the formation of flaps is a question of individual preference, modified by the location of the operation. My own peference is the circular method for amputations of the arm, fore-arm, leg and middle and lower thirds of the thigh; flaps for the fingers, hands, foot and upper third of the thigh; oval or flap for the shoulder and hip-joints, if I should be called upon to amputate at these joints; for the knee-joint, the long anterior flap, removing the patella if it cannot safely be left in the intercondyloid space; while the oval method is best for the fingers and toes at the phalango-metacarpal or metatarsal joints, always removing the heads of bones. Care should always be taken to so dispose the line of your flaps that the cicatrix shall be exposed to the least pressure after union. In the hand and foot the long flap should be taken from the palmar and plantar surfaces, for in the daily use of the hand and in walking these surfaces are most exposed to bruising pressure. At present Dr. McHatton and I have a patient who some years ago had an amputation of a portion of the foot; his surgeon (a homeopath) made his long flap from the dorsal tissue, and he has suffered ever since from a tender, inflamed foot, almost useless for walking, and will eventually require a secondary amputation to correct the mistake. In amputation of the leg and thigh the cicatrix should be nearer the posterior than the anterior surface, as the greatest pressure in the use of an artificial limb is always on the anterior surface of the stump. In the knee-joint the anterior flap gives a smooth cushion to bear the weight of the body on the end of the stump if the condyles are not removed.

We come next to the steps in the operation, and as my subject for the evening is "Amputation and Dressings," I shall simply discuss the latter in connection with the details of an amputation. A firm believer in asepsis and antiseptics, I need only to confine myself to the details of an antiseptic amputation. In the class of surgical work I do principally, railroad surgery, asepticism is an impossibility. Most of my amputations have been performed in negro cabins and in the homes of the poorer classes, where cleanliness is an unknown consideration, clean sheets and clean towels are rarely at hand for use, the bedding and furniture generally filthy, and the walls are festooned with cobwebs, and are blackened with soot, dust and dirt.

Before beginning the operation it is necessary to have everything in readiness; it is embarrassing to have to stop in the midst of an operation to look up an instrument, make a retractor, or thread a needle. You should have a number of clean towels, and a large supply of both hot and cold water. Your hands and those of the assistant should be thoroughly washed with soap and water, using a brush, then cleansed with ether and soaked in bichloride solution. Your instruments, if not already sterilized, should be placed in boiling water and then placed in trays containing warm carbolized solutions; the solution to be used as a douche during the operation prepared and in place; and the dressings to be used afterwards, cut and placed convenient. If possible you should have an operating table, the usual kitchen table will do, covered with quilts and a clean sheet if you are so fortunate as to find one, and on this the usual rubber operating cushion. The patient is etherized on the bed, and the part of the limb on which you are to operate should be shaved and scrubbed with a brush, soap and water, dried, wiped off with ether and again washed with bichloride solution; towels wrung out in bichloride solution should be wrapped around the limb both above and below the area of operation, then the patient is placed on the operating table, and the tourniquet or Esmarch's bandage applied.

These preparations having been completed, you are now ready to proceed with the operation, and for convenience I will describe the steps in the usual circular flap method. Grasping the limb with one hand, the skin should be drawn up toward the patient's body; the amputating knife should be applied so that the skin and superficial fascia can be cut entirely through down to the muscle with one clean sweep. Dissect the skin flap with clean cuts, making as few as possible. This flap should be from an inch and a half to two inches long, so as to allow for retraction. Turn back the skin cuff thus formed and cut through the muscles, blood-vessels and other tissues squarely down to the bone. Next dissect back these tissues from the bone far enough to leave a good cushion to cover the end of the bone. The periosteum should be stripped up with a dry dissector, the retractor is put on and held by an assistant, and the bone is cut squarely off by firm, long strokes of the saw. The edges of the bone, if rough, should be rounded off with the bone forceps, the ends of the nerves cut off and allowed to retract. The blood-vessels should be tied with strong catgut, cutting off short the ends of ligatures. Tie all the vessels you can before removing the Esmarch's bandage. It is a good plan before removing the constriction to squeeze the stump so as to force out any blood that may be in an untied vessel, and thus demonstrate its presence if not visible ordinarily. Remove the constriction and continue the search for bleeding points, tying and twisting the arteries as occasion indicates. The douche and sponge should now be used to wash off the wound and give a clear field for finding bleeding vessels. I prefer that this solution should be plain hot water; bichloride solutions corrode the tissues and retard their early union. All oozing of blood should be stopped before closing up the wound. In closing the wound silk sutures should be used, and in putting in the sutures you should have two rows, one deep, that is, going back about one inch from the end of the flap and embracing some muscular tissue so as to cover the bone with the latter and support the flap; the other row should be superficial, simply approximating the edges of the skin. These should be from one-fourth to one-half an inch from the flap. The drainage tube, if one is needed, should be introduced before drawing up the stitches, and it should be so placed as to protrude from the most dependent part of the wound. If you fear that suppuration will take place in the wound, it is a good plan to dust iodoform over the surface of the muscles and other tissues before finally drawing up the sutures. In drawing up the sutures they should only be tight enough to support the edges of the wound in close apposition; incurving or pouting of the edges should be avoided, as they wrinkle the edges of the wound, preventing or retarding union by strangulating the circulation in the flaps, and furthermore, the sutures will cut through, leaving suture wounds to heal up. The knots should never lie in the edges of the wound, but should rest on one side midway between the edge and the points where they come through the flap. The stump should next be washed off and flushed with the hot douche through the drainage-tube to clear out any blood or small clots that may have formed while closing up the stump. After this the dressings should be applied. Iodoform should be freely dusted over the stump, completely covering the edges of the wound. Antiseptic gauze, either iodoform or bichloride, several layers thick, should be applied, and should extend several inches beyond the stump. Over this a heavy layer of clean absorbent cotton should be placed, and the whole should be confined by the roller bandage. The gauze and cotton prevent the entrance of outside infection and catch the discharges as they come from the wound. It is a good plan, particularly if the amputation is near or just below a joint, to apply a posterior splint to the limb. This supports the limb, controls the movement of the joint, and the involuntary muscular contractions of the severed muscles, and is an aid in moving your patient when necessary. This is strongly recommended by Dr. Nicholas Senn, of Chicago, and another very useful procedure I saw him employ recently was suspension, not elevation, of the stump after amputation for several hours. This controls the circulation, diminishes oozing liable to follow, and relieves the pain in the stump. It is important that the dressings should remain undisturbed as long as possible, and yet the drainage-tube should be removed as soon as it has accomplished its purpose, that is, the removal of the early discharges from the cavity of the the stump. Usually this ends with the first forty-eight hours. Frequently the union of the wound is retarded by removing the dressings to take out the drainage-tube. To avoid this I have been in the habit, in the last few amputations, of bringing the free end of the tube entirely through the dressings, even beyond the roller bandage. This end of the tube I keep wrapped in antiseptic gauze and absorbent cotton, to prevent the entrance of air to the wound and catch the discharges, thus saving the dressings in direct contact with the wound from being soiled. The dressings on the end of the tube can be frequently changed, and when the need of the tube ceases it can be easily removed without disturbing the dressings. The dressings on the stump should never be removed without some indication that the wound is not doing well, as when there is bleeding sufficient to run through the dressings and stain the bedding, indicating secondary hemorrhage, or when the discharge of the products of inflammatory action soil the dressing excessively and run beyond these, or when fever over one hundred degrees or one hundred and three degrees is present, indicating absorption of the discharges, or when there is severe pain from too tight bandages. In this way the primary dressings may remain a week or ten days, or until you wish to remove the stitches. The most careful employment of antiseptic methods should be employed in redressing the stump. In amputations of the fingers, toes, hands and wrists, where you use no drainage, a very good plan is to paint the stump over with iodoform collodion. This hermetically seals the wound, supports the stump, and primary union goes on underneath it without risk of infection. I began this method of dressing after witnessing the good effect of collodion dressing in circumcision, and am much pleased with it.

Some of the complications and sequelæ of amputation and their treatment require a passing notice. Secondary hemorrhage may come at any time after the operation. It may be simply a severe oozing from small arterioles of the connective or muscular tissues, or from the open mouth of an artery overlooked because not bleeding prior to closing the wound, or it may be due to the slipping of the knot in the ligature, or wounding of the coats of an artery by too tight application of the ligature. The treatment may be simple or exceedingly complicated. Frequently, simple elevation of the end of the limb may control it by gravitation of the blood back to the body; or compression of the stump by tightening the bandage or moderate application of the Esmarch over the dressings for a few hours may be sufficient to arrest it. If these expedients are not sufficient, opening up the wound and ligating or twisting the bleeding vessels will be required. In applying the ligature during the operation only good surgeon's knots, and not slip knots, should be made, and only sufficient pressure to crush the internal coat of the artery should be employed; more than this destroys the artery, and is a frequent cause of secondary hemorrhage. I believe the old unyielding silk ligature, with its ends left hanging from the wound, was a frequent cause of this complication, first by cutting through the artery, and again by irritating the uniting surfaces of the wound. It is a foreign body, which is removed only after cutting through the tissues. Animal ligatures on the other hand are more yielding, they are cut short and left in the wound, and are absorbed early, leaving the artery intact without cutting through its coats. I am sure this complication is less frequent since the introduction of the animal ligatures.

Sloughing and ulcer of the flaps may be the result simply of gangrene from cutting through necrotic tissues, or it may depend on non-union from too tight sutures, improper application of the dressing, or it may be caused by the pressure of the end of the bone against the flaps when they have been cut too short. Long flaps through healthy tissues covering loosely the end of the bone, careful application of sutures so as not strangulate the circulation in the ends of the flaps and proper care in dressing, will diminish the frequency of this complication. If it does occur, it should be treated by strapping with adhesive plaster to draw the edges together, local use of oxide of zinc ointment, iodoform, powdered morphine and camphor, and later skin-grafting. If these fail or the flaps are too short, secondary amputation only remains.

Necrosis of the end of the bone and the final discharge of a piece or pieces of bone either the result of wounding the bone by the saw, or cutting through dead bone, is an annoying complication preventing the healing up of the stump. This can only be treated by removal of the bone or waiting for its final separation and discharge.

A painful, tender stump is a disagreeable sequela, the result of the skin flap growing to the end of the bone, or it may be caused by a filament of nerve being caught in the cicatrix, or it may depend on the growth of a tumor on the end of the nerve. The first can be prevented by carefully covering the bone with muscular flaps, the latter by cutting off the ends of the nerves and allowing them to retract before closing the amputation. If present, only resection of the bone, or cutting out the filament of nerve or nerve tumor, can give relief.

It is the surgeon's duty before finally discharging his patient to see that the stump is prepared for the early application of an artificial limb. The stump, immediately after recovery, tends to grow large and flabby, the joints stiff, and the muscles of the limb shrink and contract. If treatment is delayed these changes take place and it is hard to restore the limb to a condition in which an artificial limb can be worn; the early preparation of the stump and the early fitting of an artificial limb control these changes. The stump should be systematically bandaged, the parts hardened by frequent rubbing with alcohol, the joints made flexible by vigorous passive motion, and the muscles developed by massage.

